# Peer assessment of student-produced mechanics lab report videos

**DOI:** 10.1103/PhysRevPhysEducRes.13.020126

**Published:** 2017-11-01

**Authors:** Scott S. Douglas, John M. Aiken, Shih-Yin Lin, Edwin F. Greco, Emily Alicea-Muñoz, Michael F. Schatz

**Affiliations:** 1 School of Physics, Georgia Institute of Technology, Atlanta, Georgia 30032, USA; 2 Physics Education Research Lab, Michigan State University, East Lansing, Michigan 48824, USA; 3 National Changhua University of Education, 500, Taiwan

## Abstract

We examine changes in students’ rating behavior during a semester-long sequence of peer evaluation laboratory exercises in an introductory mechanics course. We perform a quantitative analysis of the ratings given by students to peers’ physics lab reports, and conduct interviews with students. We find that peers persistently assign higher ratings to lab reports than do experts, that peers begin the semester by giving high ratings most frequently and end the semester with frequent middle ratings, and that peers go through the semester without much change in the frequency of low ratings. We then use student interviews to develop a model for student engagement with peer assessment. This model is based on two competing influences which appear to shape peer evaluation behavior: a strong disinclination to give poor ratings with a complementary preference to give high ratings when in doubt, and an attempt to develop an expertlike criticality when assessing peers’ work.

## I INTRODUCTION

Peer assessment [1] has been used in classrooms across a broad range of disciplines from second-language writing [2] to conceptual physics [3], and offers the potential for instructors to administer open-ended assignments in large classes without suffering an untenable grading burden [4]. Quantitative studies of peer assessment have, to date, mostly examined peer assessment by reporting on a single peer-assessed exercise or by aggregating the results of an entire course’s worth of exercises; this is usually done to answer research questions about the overall validity or reliability of peer assessment [5–7], or questions about learning outcomes effected by peer assessment [8–10]. Our own previous work has shown the effectiveness of practice assignments in improving the accuracy of peer assessment [11], and has demonstrated quantitative improvements over time in assessment accuracy among a group of peer assessors [12]. Left unanswered are questions about *how* students’ behavior changes over time through repeated engagement with peer assessment to effect this improvement in accuracy.

In this study, we measure these changes in student assessment behavior and develop a model for these changes by examining peer assessment at the beginning and end of an introductory mechanics course. Our students participated in peer assessment several times throughout the semester-long course, each time producing and assessing content-rich lab reports in the form of 5 minute video presentations of physics experiments. We quantitatively analyze student assessments of these videos at the beginning and at the end of the semester, and then interview students to gain a qualitative understanding of their attitudes and practices. This understanding is critical for developing a more complete view of peer assessment of physics in particular, and for developing models of student critique and communication of physics concepts in general. Finally, we develop a model for student engagement with peer assessment that explains our observed changes in terms of two competing influences on students; an inclination to give high ratings (and strong disinclination to give low ratings), along with a rising recognition that instructors expect them to have high standards in assessment.

Our paper consists of the following sections: Sec. I, Introduction; Sec. II, Background, describing peer assessment generally, including a typology of its various forms; Sec. III, Laboratory Design, discussing the design of our physics laboratory activities, peer assessment process, and classroom setting; Sec. IV, Methods, which describes our statistical methods and interview procedures; Sec. V, Results, which contains our analysis of peer grades, peer ratings, and peer interviews of physics labs; and Section VI—Discussion, where we situate our results among other studies of peer assessment and discusses the framework suggested to us by our findings.

## II BACKGROUND

Peer assessment in the physics classroom is fundamentally similar to peer assessment in other fields; all peer assessment systems involve students at roughly the same level of education evaluating each other’s work. Beyond this basic similarity, though, peer assessment systems may differ widely; the assessments may be anonymous or face to face; they may provide only low-stakes formative assessment, or they may replace instructor grading entirely; participation in the system may be compulsory or voluntary; and groups, pairs, or individuals may assess the work of other groups, pairs, or individuals. The huge variety of possible peer assessment systems affords instructors great flexibility in implementation, but also cautions us against bold conclusions about peer assessment in general. Given this, researchers of peer assessment must be careful to describe the particular systems investigated in their studies, which we do in Sec. III.

While the different forms of peer assessment are numerous, the motivating benefits of peer assessment fall into four broad categories [13]:

- Logistical—A peer assessment system that produces reasonably instructorlike grades can allow an instructor to assign open-ended exercises without devoting many hours to grading.
- Pedagogical—Students’ own content knowledge can be deepened by exposure to other students’ work.
- Metacognitive—Assessment of another’s work can help a student map the boundaries of their own proficiencies and deficiencies.
- Affective—Participating in the grading process in a substantial way can make final grades seem less arbitrary and help students fulfill positive, actualized roles in a community of practice.

The logistical benefits in particular are attractive to physics instructors leading large-enrollment courses, both online and on campus; because every additional student producing work is also another peer assessor, the capacity of any peer assessment system automatically scales up with the number of students in the course. This permits the use of content-rich student exercises in courses where the assessment of such exercises would otherwise constitute an untenable burden for instructors.

Peer assessment would confer no logistical benefit at all, however, if the assessments were so untrustworthy that instructors had to spend many hours correcting them anyway. The main logistical concern of any peer assessment system is therefore validity—does the system actually produce instructorlike assessments? Previous research has demonstrated that well-designed peer assessment systems are capable of providing an adequately valid replacement for instructor grading in many fields [6,7,13]. These studies usually report validity in terms of agreement between grades given by instructors and by students to a sample of exercises. In their 2000 meta-analysis of 48 such peer assessment studies, Falchikov and Goldfinch [7] report a range of Cohen’s *d* = −0.75 to 1.25 for effect sizes comparing peer grades with expert grades, with a weighted mean *d* = −0.02 (excluding outliers), and they report a mean Pearson’s correlation coefficient of *r* = 0.69 with a range of *r* = 0.14–0.99. Both of these measures are common statistical tests of agreement, and these mean values indicate overall good agreement between peer and instructor grades.

In that meta-analysis, Falchikov and Goldfinch identified several features of peer assessment systems that contribute to validity (i.e., peer or instructor agreement), and which we paraphrase here:

- Peer assessments that involve an overall global judgment based on well-defined criteria produce better agreement than assessments which require marking several individual categories.
- Assessments of familiar academic products like essays and proofs are more likely to result in high agreement than are assessments of professional practice like simulated clinical examinations.
- Good experimental design yields higher agreement— e.g., instructors who properly reported student population characteristics and used sufficiently large populations also tended to report higher agreement.
- Assessment by large (20+) groups of peers produce worse agreement than assessments by smaller groups or by individuals.
- Student involvement with the creation of assessment rubrics tends to improve agreement [7].

Other instructors have augmented their peer assessment systems with algorithmic weighting of peer grades [3,4,14] and peer-matching procedures based on machine learning [15] to further improve accuracy. (Our own peer assessment system included one such algorithmic enhancement, but we exclude it from our current analysis in favor of looking only at the students’ raw responses.)

A subsequent meta-analysis by van Zundert *et al.* [16] examined the relationship between peer assessment procedures, student characteristics, and peer assessment outcomes. This meta-analysis identified several studies [17–19] where assessor training was shown to be associated with better peer assessment outcomes. One of our previous studies [11] also demonstrated the effectiveness of training in improving the accuracy of peer assessment, but that study only compared results between sections, not over time. Our previous study and most of the studies included in both meta-analyses were concerned with establishing the overall validity of various peer assessment systems in different classroom settings and among different populations, and so were not designed to study *how* students learn to interact with a given peer assessment system through repeated engagement—and certainly not through repeated engagement in a physics classroom, specifically.

Our current study aims to fill in this gap in the literature by exploring changes in student assessment behavior during two offerings of a semester-long introductory mechanics course featuring multiple peer evaluation assignments of physics lab report videos. One course offering was conducted entirely online; the other offering was conducted on-campus in a “flipped” classroom setting [20]. Our previous work had noted a trend toward greater student and instructor agreement over time [12]; here, we explore this trend more fully, and inform our analysis with qualitative investigations of student attitudes and practices.

## III LABORATORY DESIGN

Our “Your World is Your Lab” introductory mechanics curriculum featured four laboratory exercises designed to be completed by individual students, each centered around a physical system exhibiting a particular type of motion [21]. The sequence of systems covered by our four labs was in line with the standard introductory mechanics canon; we began with zero-net-force motion in one dimension, then motion with a variable force in one dimension, then two-dimensional motion with a central force, and finally two-dimensional harmonic oscillation.

### A Observational data

In each lab, students either received or were instructed to gather a video of a system exhibiting a specified type of motion. Students were told to use the cameras on their smartphones or the webcams built into their laptops to gather this motion data—we provided no equipment to the students. This equipment-free design was a strict necessity for an online course where students never meet in person, but it was also a convenience for our on-campus sections, since we saved time and effort by not having to set up or maintain experimental apparatus.

For the first lab (zero net force), many students elected to take videos of car traffic on residential roads, or rolled balls or cans across their desks; for the second lab (1D variable force—drag from air resistance), students selected household objects of varying shapes and densities and dropped them from a few meters’ height. We provided students with trajectory data of a simulated astronomical system for the third lab (2D central force), and gave them a video of a mass swinging on a spring for the fourth lab (2D harmonic motion). In all labs, we either provided students with data or they collected it themselves with their own tools from their own everyday surroundings.

### B Motion analysis

After collecting video motion data, students analyzed it in Tracker [22], a free Java-based video analysis tool available through the ComPADRE open source physics collection [23]. This analysis consisted of superimposing a coordinate system over the video and tracking the apparent position of the center of the moving object frame by frame, thereby producing time series data for the object’s trajectory [24].

### C Computational modeling

With this time series in hand, students then created a predictive computational model of the system in VPython [25] by modifying a provided minimally working program. Students computed the net force on the system and implemented Euler-Cromer integration of Newton’s second law; while the integration code was essentially unchanged from lab to lab, the computation of the net force in each lab involved different physical concepts such as drag, gravity, and Hooke’s law. Students then ran their computational models with parameters and initial conditions derived from their video analysis, and finally compared the model’s output with their observed trajectories. Our intent in designing this part of the laboratory process was to provide students with experience creating, testing, and validating computational models of physical systems, practices that constitute the “third pillar [26]” of modern science (along with theory and experiment) but which are often absent from the introductory physics curriculum.

### D Video lab reports

After students completed their observations, motion analysis, and computational model, they each produced a 5 minute lab report video in the style of a short contributed conference talk. Students were provided with a rubric for their lab report videos at the very beginning of the course, and were informed that they would use this same rubric to evaluate their peers’ lab report videos throughout the year (and that their own videos would be evaluated in the same manner). We also provided students with several lab report videos from previous semesters to serve as examples. Students were free to choose the style and format of their lab report videos; the vast majority of them chose to make their videos in the “screencast” format with audio narration over static or animated slides.

Students were instructed to produce a lab report video length of 5 min length, but to grant their peers an extra 1 min grace period during evaluation. Students were instructed to ignore anything after the 6 min mark in the videos they evaluated, and to deduct points accordingly. We had dual intent in setting this time limit; we wanted to set a reasonable time burden for students in both producing and evaluating their lab report videos, and we wanted to give them experience with the strict time limits characteristic of presentations at academic conferences. As with computational modeling, the formal presentation of scientific concepts within a given time limit is another practice expected of professional scientists but often absent from the physics classroom [27].

### E Peer assessment process

At the end of each lab, after students submitted their lab report videos, they began a peer assessment process that comprised a “training” phase followed by an “evaluation” phase. All students were required to complete both phases of peer assessment themselves, but we made no attempt to ensure that students worked alone.

We designed the training phase to improve the accuracy of assessments, and to increase student buy-in to the assessment process. This phase comprised four lab report videos from previous semesters, selected and assessed by instructors. During training, each student viewed each video online, assessed it, and submitted the assessment electronically, after which they were presented with the detailed instructor assessment for that video. We directed students to compare their own assessments with the instructors’ before proceeding to the next training video.

The first two training videos in each lab were for practice only, and carried no grade. The latter two training videos carried a grade incentive; students received a small grade penalty for assessments with ratings far from the ratings given by instructors. A fifth, final training video disguised as a peer video was presented to students during the evaluation phase to further ensure the integrity of the assessment system [28].

During the evaluation phase, students were assigned three random peers’ lab report videos (and one disguised training video), and asked to evaluate them. The students were also assigned their own videos for self-evaluation. Students could assess all five videos at their leisure, in whatever order they chose.

### F Rubric

Each peer or expert grade was calculated from a rubric consisting of five items rated on a five-point poor-to-excellent rating scale. Each possible rating was assigned a numerical score. During peer grading, our peer assessment system showed students the overall numerical grade corresponding to their selected set of ratings. Students were given an unlimited number of opportunities to revise their ratings before the assignment deadline.

An early version of our rubric excluded any assessment of the lab report video’s production quality. The instructors believed that the rubric should reflect the goals of the course, and that developing good production quality (visual effects, audio cues, overall attractiveness, etc.) was a relatively unimportant goal compared to the development of students’ physics content mastery and argumentation. However, it was later determined that production quality was still an important consideration in students’ minds, and could potentially influence students’ ratings of unrelated rubric items if they were not given the means to specifically express their opinions about it. In the final version of the rubric (used for this study), we accommodated this consideration by including a production quality item on the rubric, but assigning it relatively fewer points than the other four items.

See the Appendix for the full rubric used in this study.

### G Topping typology

Topping [29] provides a comprehensive typology of peer assessment systems. Peer assessment as practiced in our Your World is Your Lab introductory mechanics course had the dual objectives of course scalability and student improvement in critique and communication of physics. Our peer assessment system *per se* focused on quantitative, summative assessment, with some qualitative assessment provided through peer comments and some formative assessment during small-group rehearsals. Lab report videos were the objects of assessment. Peer assessment substituted for instructor assessment, and contributed to the assessee’s final course grade. Assessment was one way, unsigned (the assessors were unknown to the assessee, but the assessee was known to the assessors), with no face-to-face contact during assessment. Assessors were in the same course as the assessee, which implies similar ability but not necessarily same year of study. Groups of assessors assessed individual assessees, but assessors all worked independently and were unknown to each other. Assessment took place outside of the classroom, was compulsory, and counted for course credit.

### H Classroom practices

#### 1 Spring 2014—On campus

In Spring 2014, we administered peer assessment in a flipped [20] on-campus classroom context at Georgia Tech. The 338 students involved were enrolled in two experimental sections of a large-enrollment Physics I course (PHYS 2211). This course used the Matter and Interactions textbook [30].

For the two experimental flipped sections, we replaced live lectures with online video lectures from the Georgia Tech Your World is Your Lab curriculum [31]. Class time originally devoted to lecture was instead spent on group work and problem solving.

Each student was required to conduct their own experiment and submit their own video lab report, but we did not deliberately discourage students from working together. During class time, students met in small groups (~4 students) to present and receive feedback on their inprogress lab exercises and lab report videos.

If students had a dispute with the peer grade they received for their lab report videos, they could avail themselves of a regrade by a teaching assistant (TA).

#### 2 Summer 2016—Online

In Summer 2016, we administered peer assessment entirely online in a Summer Online Undergraduate Program (SOUP) Physics I course. The SOUP course used the Matter and Interactions textbook and the Your World is Your Lab online lecture videos, just like the on-campus flipped course. The entire course was conducted online; students met online periodically for group work and TA-led question and answer sessions. 21 students participated in the SOUP.

Each student was required to conduct their own experiment and submit their own video lab report, but as in the on-campus course, students were not actively discouraged from working together. Once per lab, students met online through Google Hangouts [32] in small groups (~4 students, plus one TA) to present and receive feedback on their in-progress lab exercises and lab report videos.

As before, if students had a dispute with the peer grade they received for their lab report videos, they could avail themselves of a regrade by a TA.

### I Student interviews

We conducted five student interviews after the final lab of the SOUP to inform our quantitative analysis with an investigation of student attitudes and reasoning. We administered the interviews online over Google Hangouts, and recorded video of each interview. Each interview was conducted by the same researcher and lasted roughly an hour. The interview began with a think-aloud exercise [33] where the student performed a warm-up problem and then watched and assessed a lab video in the same manner as during the course. The think-aloud portion of the study was immediately followed by a semistructured interview with follow-up questions.

During the lab video assessment, the student’s computer screen was recorded, capturing the lab video itself and everything the student typed. At all other times, video of the student’s face was recorded. Transcripts were made for each interview.

## IV STATISTICAL METHODS

Cheating and guesswork were concerns for our peer assessment system, especially for the training-phase videos. Before performing any statistical analysis, we scrubbed our data set of any student assessments we deemed suspicious. For videos for which clickstream data was available, we removed all assessments from students who were not recorded as having viewed that video. For videos included in the training phase, we removed all assessments from students who were in exact item-by-item agreement with the expert assessment for that video. This scrubbing process likely resulted in the removal of some legitimate assessments; nevertheless, this process successfully eliminated an anomalous peak at zero student-expert score difference in Fig. 2(b), and any more forgiving process for determining “suspicion” would have required an unacceptable number of subjective judgment calls. Overall, the scrubbing process removed 413 of 3065 assessments originally intended for Fig. 2(b).

For our quantitative analyses, we compared distributions of grades and ratings with a nonparametric Kolmogorov-Smirnoff two-sample test [34] (KS test) with *α* = 0.05. In cases where the KS test yielded a statistically significant difference between distributions of grades, we reported effect size using Cohen’s *d* [35]. In Table I, we compare variances with a Levene test [36], but we report standard deviations instead of variances for ease of interpretation. In all cases, error bars represent 95% confidence intervals calculated from a 10^4^-sample random uniform bootstrap [37].

**Table I:** Differences between peer and expert grade for all peer grades of two groups of physics lab report videos (Group A, 204 randomly selected peer videos, 3 peers per video: Group B, 20 training-phase videos, 338 peers/video). Distributions of differences are compared with a KS 2-sample test, with effect sizes calculated by Cohen’s *d*. Variances are compared with a Levene test. Standard deviations are reported instead of variances for clarity.

Group		Pre	Post	Significance	Effect size
A	*N*_pairs_	305	261		
	Mean	12.38	7.30	*p* ≪ 0.01	*d* = −0.51
	Std	9.23	10.86	*p* = 0.05	⋯
B	*N*_pairs_	1489	1163		
	Mean	11.94	6.33	*p* ≪ 0.01	*d* = −0.53
	Std	10.57	10.57	*p* = 0.29	⋯

**Fig. 1:**
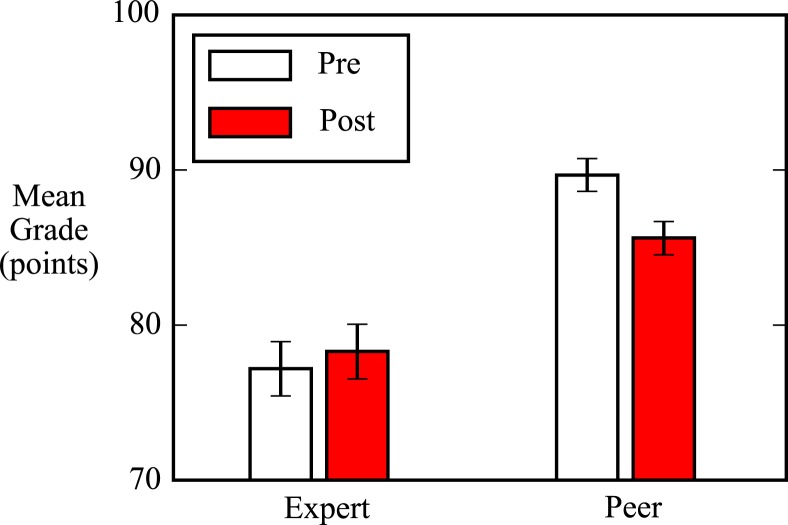
Peer assessments of physics lab report videos taken from the beginning (pre) and end (post) of the semester show that the mean peer grade becomes significantly lower and closer to the mean expert grade by 5 points, about half a letter grade on a standard 100-point grading scale (*p* ≪ 0.01, Cohen’s *d* = −0.45). A two-sample KS test shows distributions of expert grades do not significantly change (*p* = 0.86). Means are determined from 565 peer grades of a sample of 204 physics lab report videos selected from the first and last labs of the semester, along with expert grades for each of those videos. Error bars show 95% confidence intervals.

Discrepancies in reported *N* values arose from students withdrawing from the course, neglecting to grade all of their assigned videos, or neglecting to give a rating for all five rubric items for a given video. For example, in Fig. 1, 204 video lab reports assigned to three peers each yielded only 565 complete peer grades, not 612.

## V RESULTS

Here, we compare expert and peer grades of physics lab report videos selected from the beginning and end of the semester. All grade data are gathered from the Spring 2014 on-campus session unless otherwise stated. We find that the difference between student and expert grades diminishes over the course of the semester, and we follow this trend in our data through three increasingly fine-grained levels of quantitative analysis.

### A Peer and expert grades

Our highest-level examination of student and expert evaluation behavior reveals that our students gave significantly lower grades to their peers’ lab reports on average at the end of the semester (“post”) than they did at the beginning (“pre”), while average expert grades for those same lab reports held steady. Mean peer pre and post grades (respectively, 90% and 86%, *p* ≪ 0.05, Cohen’s *d* = −0.45) were around a full letter grade higher than mean expert pre and post grades (respectively, 77% and 78%, *p* > 0.05). Figure 1 shows the mean peer and expert grades for 204 lab reports submitted by peers at the beginning and end of the semester, each of which was assigned to three different peers for grading.

That the mean expert grade of students’ lab report videos did not change significantly over the course of the semester should not be taken as evidence that the students did not improve in their physics understanding or in their laboratory skills. Unlike survey instruments, which are typically administered unchanged between pre and post, the labs were for-credit assignments situated in the natural progression of an introductory mechanics course. The last laboratory assignment of the semester involved a more complicated system than did the first (two-dimensional harmonic oscillation vs one-dimensional constant-velocity motion, respectively). The last lab also required students to perform an analysis of mechanical energy, which the first lab did not. Since the physics content and methodological requirements of the last lab were more difficult than those of the first, we view the stability of expert grades as evidence that students were overall able to stay on par with increasing instructor expectations.

The decline in mean peer grade indicates that peer evaluation behavior did change meaningfully over the course of the semester, and the relative stability of expert grades suggests that expert evaluations can serve as a useful baseline against which to compare peer grades.

### B Peer and expert grade differences

**Fig. 2:**
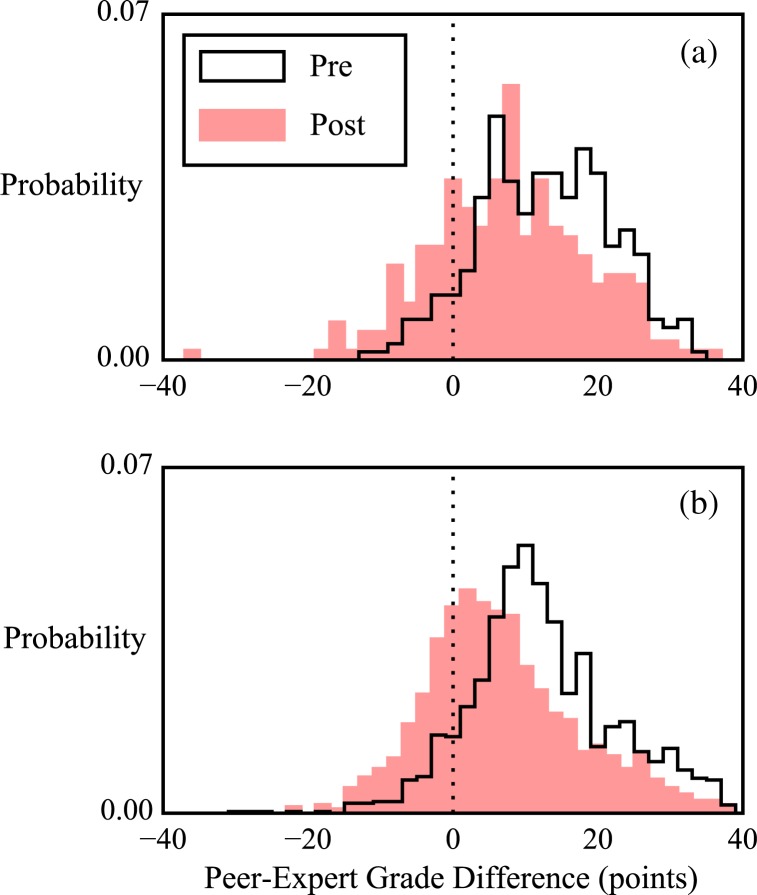
Distributions of differences between peer and expert grades show that peer grades become more accurate, but not more precise relative to expert grades. Two distinct grading schemes are considered: (a) In grading scheme one, many peer videos are each graded by few peers. (b) In grading scheme two, 20 training-phase videos are each graded by all peers. The mean grade differences shift significantly toward zero over the course of the semester, indicating peer grades increase in accuracy. The widths of the distributions do not change significantly, indicating peer grades do not become more precise. See Table I for *N* values and full tabulation of results.

Our next level of analysis involves matching peer grades to expert grades and taking the difference, rather than just comparing the means of the overall distributions of grades. We examine two distinct data sets; an *N* = 566 set of paired peer-expert grade differences for 204 randomly sampled videos graded by 3 peers each [Fig. 2(a)], and an *N* = 2652 set of paired peer-expert grade differences for 20 training-phase videos graded by all peers [Fig. 2(b)]. In Fig. 2, high peer-expert agreement would be indicated by narrow distributions centered on zero. Overall, the distributions of grade differences in both data sets shifted significantly toward zero over the course of the semester, but they did not significantly narrow. We therefore conclude that peer grading became more accurate over the course of the semester, but not more precise. See Table I for full tabulation of results.

We hypothesize that these results may indicate peers’ experiences during the semester taught them that experts tended to give lower grades than peers did (accounting for the shift toward zero in Fig. 2 and the lowering of the mean peer grade in Fig. 1), but did not help them develop any further expertlike rating behavior (accounting for the lack of narrowing in Fig. 2).

To inform this hypothesis, we examined matched peer-expert grades at a yet lower level by breaking them into their constituent poor to excellent ratings.

### C Peer and expert ratings

**Fig. 3:**
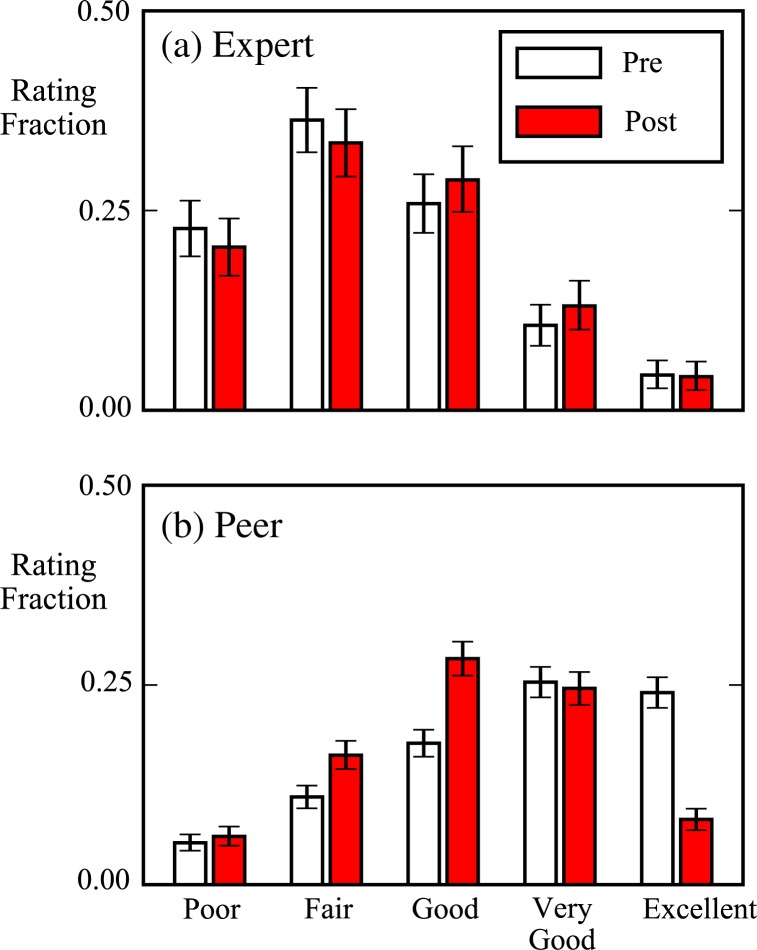
Expert rating distributions (a) do not change significantly from pre to post, while peer rating distributions (b) change dramatically. Five ratings on a five-point poor to excellent scale determine the numerical value of each peer or expert grade. Excellent changes from being among the most frequent peer ratings to among the least frequent, and good ratings become substantially more frequent. Figure shows 2820 peer ratings of a sample of 204 physics lab report videos selected from the first and last labs of the semester, along with expert ratings for each of those videos. Error bars show 95% confidence intervals.

We compare the distributions of ratings underlying the grades given by peers and by experts at the beginning and end of the semester (Fig. 3).

#### 1 Ratings overall

We find that the overall distribution of expert ratings is relatively stable across the semester. This means that expert ratings can, like expert grades, serve as a useful basis of comparison when describing student ratings. Experts give “fair” ratings most frequently, and give comparatively few “excellent”’ ratings, in contrast to peers who begin the semester giving more “good” and excellent ratings than any other rating.

By the end of the semester, the distribution of peer ratings had shifted substantially. The fraction of peer excellent ratings greatly diminished, and the fraction of peer good ratings greatly increased. No such trend was present among poor and fair ratings at the lower end of the rating scale; those rating fractions exhibited insignificant or relatively small changes, respectively. The “very good” rating fraction also did not change significantly.

#### 2 Ratings for physics-content rubric items

**Fig. 4:**
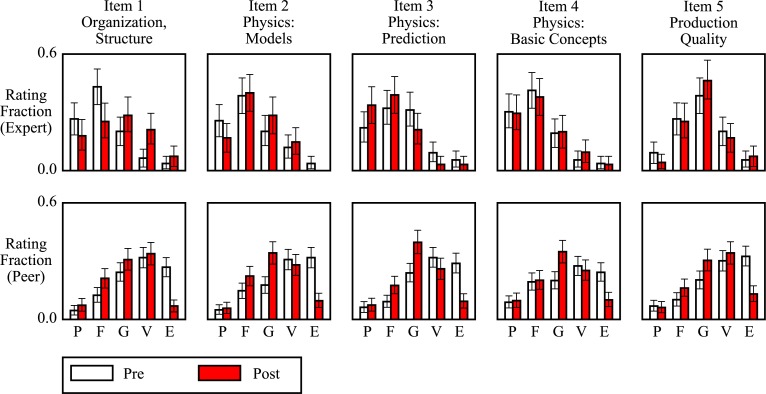
Peer ratings for physics-content rubric items (2, 3, and 4) show a greater pre-to-post decline than peer ratings for nonphysics-content rubric items (1 and 5). Consistent among all rubric items was a large decline in peer excellent ratings. Expert ratings were relatively stable across rubric items, with the exception of item 1 (organization and structure). Figure shows 2820 peer ratings of a sample of 204 physics lab report videos selected from the first and last labs of the semester, along with expert ratings for each of those videos, sorted by the rubric item to which each rating was assigned. Error bars show 95% confidence intervals. See Table II for a tabulation of comparisons.

At our final level of quantitative analysis, we compared the same sets of peer and expert ratings as above, but broken down by rubric item (Fig. 4). The five items on the rubric comprised three physics-content and two nonphysics-content items, encompassing the whole range of instructor expectations for lab video production. Items 2, 3, and 4, the physics-content items, asked the reviewer to assess, respectively, the author’s explanation of the physical model relevant to that lab, their discussion of their computationally simulated predictions versus their observations, and their overall grasp of basic physics concepts. For the full text of the rubric, see the Appendix.

Among the physics-content items, the peer ratings changed more than the expert ratings over the course of the semester, in line with our other findings regarding the relative stability of expert grades and ratings. In all three cases, the value of the two-sample KS test statistic (a proxy for effect size) for pre-to-postcomparisons of student ratings are larger than for pre-to-postcomparisons of expert ratings.

As measured by the KS test statistic, the peer and expert distributions became more similar to each other over the course of the semester in items 2 (physics models, *D*_pre_ = 0.47, *D*_post_ = 0.29) and 4 (general physics concepts, *D*_pre_ = 0.43, *D*_post_ = 0.38), but not item 3 (prediction discussion, *D*_pre_ = 0.46, *D*_post_ = 0.48).

In all cases, the most common expert rating for physics-content items was fair, and the proportion of peer excellent ratings fell over the course of the semester. In all pre cases, the most common student rating for physics-content items was very good or excellent, while in all post cases the most common student rating was good.

#### 3 Ratings for nonphysics-content rubric items

Items 1 and 5, the nonphysics-content items, asked about the organizational and structural quality of the presentation, and the audio and visual quality of the video *per se*, respectively. For the full text of the rubric, see the Appendix.

The expert ratings distributions for both nonphysics-content items buck the trends of the physics-content items. Item 1 (organization and structure) shows a relatively large pre-to-post change in expert ratings distributions compared to the peer ratings (*D*_expert_ = 0.27, *D*_peer_ = 0.20), and the most common expert rating for this item changes from fair’ to good’, unlike any of the physics-content items.

Item 5 (production quality) shows a stable expert ratings distribution with a substantial peak at good, unlike any other rubric item. Among both nonphysics-content items, as measured by the KS test statistic, student ratings distributions became closer to expert ratings distributions than for physics-content items.

### D Interviews

After we analyzed our Spring 2014 student ratings, we interviewed five students who took our course online in Summer 2016. We aimed to explore the attitudes expressed by students about the peer assessment process, the laboratory exercises, and instructor expectations, and to explore how those attitudes changed over time.

We found two recurring, related themes among the student interviews. The first was the idea that experts had critical or harsh attitudes toward grading, while students’ own attitudes were “nice,” especially toward the beginning of the semester. The second was that low or bad ratings required a higher level of confidence and authority on the part of the rater than did higher ratings, and that the instructors’ greater experience and physics content knowledge endowed them with more confidence and authority than students.

One student described her change in rating behavior over the semester in terms of a reevaluation of her own inclination toward niceness, while mentioning her uncertainty about being critical (all quotes edited to remove dysfluencies):

Interviewer You’re describing the experts having a different set of knowledge about physics: more knowledge about physics, a better ability to attend to physics details. But overall, if you had to describe it generally, what do you think is the difference between how the experts and how you evaluate these videos?

Student 1 I think now, I would say I’m a lot more accurate, I guess, in terms of like comparing [my ratings with] the experts. I think the first time I did [an assessment], I think I was like being very nice, and I gave a lot of excellents. And then I realized, like, “Oh, that was like not what it was at all!” So I think over time I’ve realized to be more critical toward the video, I guess. Cuz at first I was like “Oh, yeah, that was good, that was good!” And then like… as I’ve gone on, I’ve like gotten more used to being very critical towards like certain things that people say… I guess, like, if people say something wrong, it’s not—I think in the beginning, I was like, “Oh, well, maybe they just like said the wrong thing.” But like… they said the wrong thing, so I should mark them down.

Interviewer So at the beginning you said you thought—at the beginning of the semester you, uh, felt like you were being nice to your fellow students. Why did you feel like being nice to your fellow students?

Student 1 Well, I also think I was like… not sure how harsh to be. [laughs] I guess!

Interviewed students often attributed the differences between peer and expert attitudes to the greater physics knowledge possessed by the experts. With respect to the first theme—the relative harshness of expert ratings—interviewed students described the experts as being able to attend to and identify smaller and subtler “physics errors” than students, since the experts knew more about physics. Students described themselves as not possessing the knowledge necessary to notice these errors, and so they gave higher ratings to their peers’ videos than they would have if they had been able to notice the errors. Follow-up questions revealed the students’ usage of physics error to mean something like a misconception, i.e., a flawed or undeveloped characterization of a physics concept, rather than a mathematical or notational error *per se*.

One student made a particularly clear expression of her own inexperience relative to the instructors, in the context of describing the difference between her ratings and the instructors’ ratings:

Student 2 I guess… the difference between [our ways of] grading comes down to like, I’m a student, I don’t know as much as a professor does. And a professor has encountered a lot more, like, training, and a lot more experience to actually grade someone else’s work. So, they’re a bit more meticulous. And I mean, a professor is teaching a subject so that another person learns the subject. I—I’m learning it, so I’m not as meticulous as someone else, because I’m also trying to grasp the concepts.

Finally, some interviewed students explained their own nice attitudes by describing a feeling of identification with their peers—as one student put it, the author of any given peer video “was in the same position [she] was,” and they were “all trying to get a good grade in this class.” Other students expressed camaraderie in different ways, such as “we’re all at the same level,” or by framing the idea in terms of mutual respect for effort; one student said it was important to take grading seriously “because they took time just like I took time to make my video.”

## VI DISCUSSION

### A Generalizability of interview results

The students we interviewed in 2016 took the course in a different context than did our 2014 students, even though the curriculum and peer assessment system were unchanged. The sole 2016 section comprised 21 students and was conducted entirely online, while the 2014 sections comprised 338 students and had online and on-campus components.

**Fig. 5:**
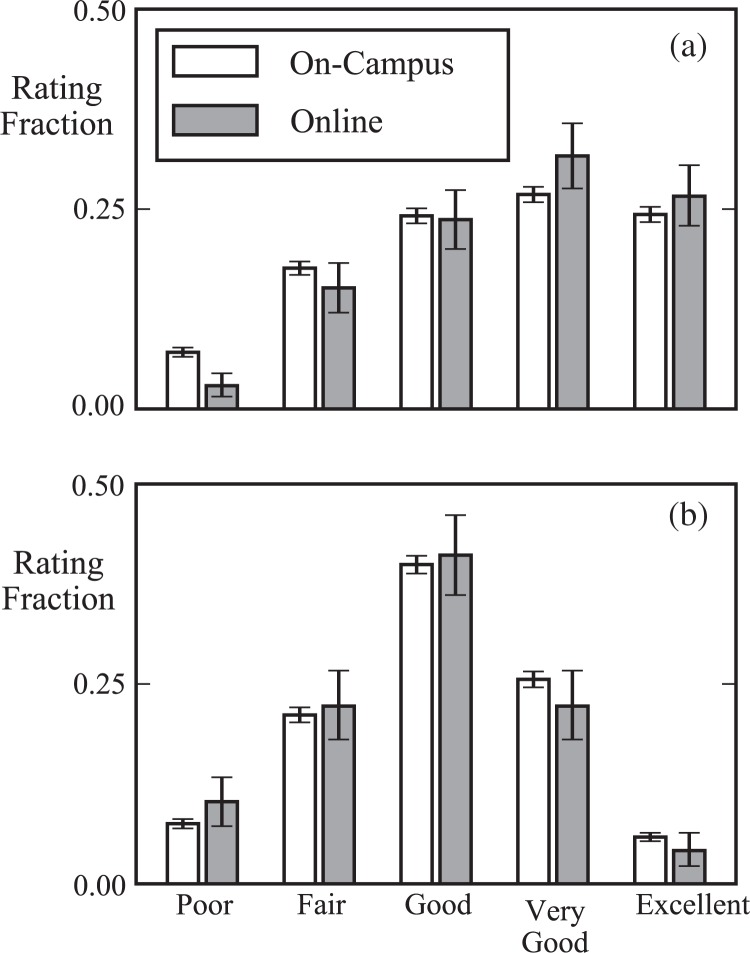
Peer rating distributions in two different classroom contexts—on campus and online—show the same pre (a) and post (b) trends. The on-campus offering was conducted in 2014, and comprised 338 enrolled Georgia Tech students. The online offering was conducted in 2016, and comprised 21 enrolled Georgia Tech students. Peer ratings of 10 videos evaluated by students in both course offerings are shown. The similarity of the distributions indicates good overall repeatability, and suggests that the students in both classroom contexts are generally comparable with respect to their peer evaluation behavior. Error bars show 95% confidence intervals.

Nevertheless, the peer rating distributions of the 10 pre and post videos rated in common by all online and on-campus peers show a very high degree of similarity (see Fig. 5), and appear to recapitulate the trends we found in the on-campus peer ratings (compare Fig. 5 to Fig. 3). We therefore believe that the experiences of both cohorts with respect to peer evaluation are comparable, and that the interviews of the online students can yield broadly applicable insights into the peer evaluation process among both cohorts.

### B Our Peer assessment system compared to others

To situate the results of our study in the existing literature, we compared our results to the findings of Falchikov and Goldfinch’s [7] meta-analysis of 48 peer evaluation studies. This meta-analysis reported a range of 0.75–1.25 for effect sizes comparing peer grades with expert grades for a matched set of exercises, with a weighted mean effect size of 0.02. The authors of this meta-analysis cite the close-to-zero mean *d* as evidence that peer grades agree well with expert grades, on average.

Our own student and instructor effect sizes at the beginning (pre) and end (post) of the semester were *d*_pre_ = 1.33 and *d*_post_ = 0.83, respectively [38]. These effect sizes provide additional support for our conclusion that peer and expert agreement increased over time, but they also place our study at the very high end of the effect size range found by the meta-analysis. High positive values of effect size mean that our peers gave significantly higher grades than did the experts, and though this effect diminished with time, it was still large at the end of the semester.

In their meta-analysis, Falchikov and Goldfinch also compared correlations between peer grades and expert grades, finding a mean Pearson’s correlation coefficient of *r* = 0.69 with a range of *r* = 0.14–0.99, concluding that the relatively high mean r further supported the conclusion that peer and expert grades agreed reasonably well [7]. Here, high correlation indicates high agreement between peers and experts. Our own data shows a peer and expert correlation *r*_pre_ = 0.51 and an *r*_post_ = 0.25, which would appear to indicate a declining peer and expert agreement over time, in contradiction to our other findings. However, we believe this interpretation is inapplicable to our data. Our peers’ pre grades were at the very top of the grade scale and thus constrained by the grade ceiling at 100 points; this ceiling effect forced a narrowing of the peer grade spread at higher values of expert grade, thereby leading to an artificially high correlation coefficient. The post grades were on average lower than the pre grades, and thus not subject to a ceiling effect. We therefore disregard our measured decline in *r* and again simply interpret our relatively low correlations as indicative of low peer and expert agreement compared to other peer assessment studies.

This comparatively low agreement deserves examination. Our own peer assessment system featured two prominent factors identified by the authors of the meta-analysis as contributing to low peer and expert agreement. First, the authors conclude that peer assessment studies which have students assess “academic products and processes” of the sort they “have experienced for much of their formal education” tend to have higher agreement than studies where students assess “professional practice which requires them to learn a new set of skills” [7]. The lab report videos our own students produced were novel products, intended to include some practical elements of professional scientific communication. Students had to learn video production techniques and new communication skills and were unlikely to have previously conducted a formal critique of such work in the classroom, so it seems fair to characterize our own peer assessment study as an assessment of “professional practice” as defined by Falchikov and Goldfinch.

Second, Falchikov and Goldfinch conclude that studies where students feel more “ownership” of the assessment criteria (e.g., where students are directly involved in developing the grading rubric) tend to exhibit higher agreement [7]. Our own rubric development process did not involve the direct input of students.

Given the presence of both of these features, it is perhaps not surprising that our own study produced peer and expert agreement on the low end of the scale compared to other peer assessment systems. However, our research goals reach beyond establishing whether our peer assessment system is capable of producing valid grades; we aim to examine the changes in student behavior effected by our peer assessment system.

### C Assessing physics content vs nonphysics content

We take the relative stability of expert ratings overall as evidence that, in general, students were able to keep track with instructor expectations for lab report videos. However, not all rubric items showed the same trends in expert ratings; item 1 (a nonphysics-content item assessing organization and rhetorical structure) showed the largest statistical pre-to-post change in expert ratings distributions, and was also the only rubric item to see a change in the modal expert rating (fair to good). Taking expert ratings as “ground truth,” then, this suggests that students were able to improve the organization and rhetorical structure of their lab report videos more than they were able to improve their command of physics concepts or the production quality of their videos, relative to instructor expectations.

This is a positive result: we developed these laboratory activities and our peer review system with the goal of giving our students authentic experiences with physics communication and critique, and finding an improvement in the structural and rhetorical quality of the videos suggests that these activities are indeed helping students to develop some more expertlike physics communication skills.

Expert ratings of the physics-content items, on the other hand, suggest that students did not exceed instructor expectations for physics content, but rather kept pace with them or fell slightly behind (as was the case with item 3—prediction—though whether this is a general result about prediction or if it is attributable to the specifics of the Lab 1 and Lab 4 prediction tasks is difficult to say).

Finally, all our analyses consistently indicate that student ratings move closer to expert ratings over the course of the semester, even within individual rubric items (though some items show more improvement than others—see Table II).

**Table II:** Results of comparisons between peer and expert ratings of two groups of physics lab report videos (204 videos, 3 peers per video, 5 ratings per peer per video). Items 2, 3, and 4 assess physics content; items 1 and 5 do not. Distributions are compared with a KS 2-sample test, with results reported by the KS test statistic *D* [34]. *D*_peer_ compares the pre peer ratings with the post peer ratings, and likewise *D*_expert_. *D*_pre_ compares the pre peer ratings with the pre expert ratings, and likewise *D*_post_. *D* ∈ ℝ, 0 ≤ *D* ≤ 1. *D* (which does not measure central tendency) is not equivalent to Cohen’s *d*, but *D* does provide a consistent way to compare whether one pair of distributions is “more different” or “more similar” than another pair: *D* = 1 when two distributions do not overlap at all (maximum difference), *D* = 0 for identical distributions (maximum similarity), *D* varies continuously in between.

	Item 1	Item 2	Item 3	Item 4	Item 5
*D*_peer_	0.20	0.25	0.25	0.17	0.19
*D*_expert_	0.27	0.09	0.19	0.04	0.06
*D*_pre_	0.53	0.47	0.46	0.43	0.37
*D*_post_	0.15	0.29	0.48	0.38	0.23

These quantitative results, taken together, indicate that the introduction of video lab reports and peer assessment improved students’ physics communication and critique skills while complementing (or at least not significantly interfering with) their conceptual physics development. In this respect, these physics lab exercises would appear to have fulfilled our instructional goals. Over the course of the semester, we found students were consistently able to

- create lab report videos which kept pace with experts’ expectations for physics content,
- create lab report videos which improved substantially in organization and rhetorical structure, and
- improve their ability to assign expertlike ratings to their peers’ lab report videos, as evidenced by improved agreement with expert ratings.

This improvement in student ratings does not, however, necessarily imply the development of more expertlike attitudes and practices among students. Students may have achieved greater agreement with expert ratings through, e.g., the adoption of simple heuristics like deliberately giving fewer excellents, or by attending to surface features of peers’ explanations that are correlated with, but not actually demonstrative of, expertlike physics understanding. To understand the attitudinal and cognitive changes undergone by students in peer assessment, then, we must develop a qualitative model for student engagement with peer assessment.

### D A model for student engagement with peer assessment

We believe that a model helpful for understanding students’ evolving engagement with peer assessment should address metacognition and participation in a community of practice.

All physics work involves metacognition to some degree, but the sort of tasks involved in peer assessment confront students with especially stark decisions about how confident they are in their own knowledge. If any part of a student-produced work seems confusing or flawed to a student assessor, the assessor must decide whether the work is faulty or if their own understanding is faulty—a metacognitive question. When a student encounters confusing passages in a textbook or exam question, on the other hand, the general presumption of novice students is that the passage is correct and their own understanding is at fault. Furthermore, the stakes of metacognition in peer assessment are especially high compared to other metacognitive tasks. Consider the case of a student assessing another student’s explanation of Newton’s second law—not only must the assessor decide how good the explanation is, she may also have to decide whether she is confident enough in her assessment to assign a bad grade to her fellow classmate.

Likewise, all physics work is conducted in a community of practice, but students’ understanding of their role in that community (and the norms and practices of that community) are especially pertinent to peer assessment. The role of an anonymous peer reviewer is unlike any other role we ask students to perform in our introductory physics courses, and so student adaptation to that role is of particular interest to us as researchers. We also provide extensive training videos and grade incentives to guide students to internalize instructorlike sets of norms regarding physics content and the different ratings on the rating scale. The expectations and practices associated with normative judgment of peers’ performance are unique to peer assessment, and deserve special attention in our theoretical model.

We reviewed our findings with these two frameworks in mind, and found three major themes relevant to community practice and metacognition:

- Student interviews revealed an inclination to give high ratings and a strong disinclination to give low ratings.
- Quantitative analysis revealed a decline in high ratings but no corresponding rise in low ratings.
- Student interviews suggested a relationship between self-assessed proficiency in physics content and the justifiability of giving low ratings.

With these themes, we can outline a model of students’ evolving engagement with peer assessment of physics communication in terms of two competing influences: an attitude toward the rating scale that includes an initial inclination to rate peers highly, and an enduring reluctance to assign low grades born of uncertainty and a self-assessed lack of physics knowledge, and a rising recognition that experts expect students to have high standards when assigning ratings. This latter influence stands in tension with the former, and students working to resolve this tension could plausibly yield the changes in ratings we observe.

For example, among the peer ratings, excellent declined in frequency, good increased, and the other three ratings did not significantly change from the first lab to the last. In our proposed model, students begin their first act of peer assessment with the expectation that high ratings are readily achievable by their peers and may be given freely, while low ratings require extra care and confidence and should not be given lightly. This inclination toward leniency may be related to the sense of sympathetic camaraderie students bear toward each other, as mentioned in our interviews.

Over the course of the semester, as students learn more about instructor expectations regarding lab reports and gain more experience in their roles as peer assessors, students begin to consider ratings at the high end of the scale loftier and less achievable than they once did. As they gain familiarity with the rubric and with instructor expectations for the lab reports, they also learn to attend to their peers’ videos with a more critical, scrutinizing attitude. We would expect these factors to lead to proportionately fewer high ratings as the semester goes on.

On the other end of the rating scale, we propose that students persist in the justifiable belief that low ratings deserve special care, that low ratings should only be assigned when they are confident that the work deserves a low rating, and that confidence in rating is born of expertise with physics content. Students we interviewed consistently reported an awareness that they were learning physics at an introductory level, and that their physics content knowledge was much less than that of an instructor—it is unlikely that a student would begin to consider themselves an expert after only a semester of introductory-level instruction, so we would predict that the proportion of low ratings would not change very much over the course of the semester.

Our particular data cannot definitively confirm any one model of student behavior in peer assessment, and the predictive value of our model needs to be demonstrated through future work. We might expect, for example, to find very different initial distributions of ratings among students in a less cooperative classroom culture (e.g., a strictly curved class where students compete for a fixed number of A’s, or at an institution where much more emphasis is placed on class rank). We may also expect to find a different evolution at the low end of the rating scale among peers in upper-level physics courses. Our model, such as it is, serves mainly as a useful and suggestive interpretive tool to inform further research. Specifically, we aim to investigate the connection between peer assessment and other classroom behaviors (such as video-watching behavior and the way students present physics concepts in their videos) to inform a more general model of student behavior in peer assessment. Further study with a standardized conceptual instrument (such as the Force and Motion Conceptual Evaluation [40]) should also help shed light on the effects of these lab exercises and peer assessment on physics concept development in particular.

## APPENDIX: RUBRIC

The full rubric used in this study for peer assessments, as it appeared to students.
